# Adaptive Repetitive Control of A Linear Oscillating Motor under Periodic Hydraulic Step Load

**DOI:** 10.3390/s20041140

**Published:** 2020-02-19

**Authors:** Xinglu Li, Zongxia Jiao, Yang Li, Yuan Cao

**Affiliations:** 1 School of Automation Science and Electrical Engineering, Beihang University, Beijing 100191, China; lixinglu@buaa.edu.cn (X.L.); leeyoung303@163.com (Y.L.); caoyuan@buaa.edu.cn (Y.C.); 2 Ningbo Institute of Technology, Beihang University, Ningbo 315800, China

**Keywords:** hybrid electrohydraulic pump, repetitive control, adaptive control, linear oscillation motor, step load

## Abstract

A linear oscillating motor has a direct and efficient linear motion output and is widely used in linear actuation systems. The motor is often applied to compact hybrid electrohydraulic actuators to drive a linear pump. However, the periodic switch of the rectification valve in the pump brings the hydraulic step load to the linear motor, which causes periodic oscillation waveform distortions. The distortion results in the reduction of pumping capacity. The conventional feedback proportional-integral-derivative control is applied to the pump, however, this solution cannot handle the step load as well as resolving nonlinear properties and uncertainties. In this paper, we introduce a nonlinear model to identify periodic hydraulic load. Then, the loads are broken up into a set of simple terms by Fourier series approximation. The uncertain terms and other modeling uncertainties are estimated and compensated by the practical adaptive controller. A robust control term is also developed to handle uncertain nonlinearities. The controller overcame drawbacks of conventional repetitive controllers, such as heavy memory requirements and noise sensitivity. The controller can achieve a prescribed final tracking accuracy under periodic hydraulic load via Lyapunov analysis. Finally, experimental results on the linear oscillating motor-pump are provided for validation of the effectiveness of the scheme.

## 1. Introduction

Compact hybrid electrohydraulic actuators have been widely reported in literature in recent years [1]. The actuator consists of a pump driven by piezoelectric stacks. Raising the oscillation frequency can boost flow rate, but the bandwidth of the rectification valve limits the frequency in a certain range. The inherent low amplitude of the piezoelectric stack hinders improvement of the flow rate [2,3]. The linear oscillating motor can achieve the oscillation motion of a relatively large amplitude and is considered as the driver of electro-hydrostatic systems. Liang et al. [4] developed a tubular linear oscillating motor to use in the electro-hydrostatic system. Wang et al. [5] optimized the linear oscillating motor, which achieved a high thrust and eliminated the eddy current effect. The oscillation reached ±5 mm stroke and 30 Hz frequency. The thrust force reached 500 N. Based on the linear oscillating motor, Li et al. [6] developed the linear drive collaborative rectification structure pump (LDP). Jiao et al. [7] invented the linear drive electro-hydrostatic actuator and experiments were conducted. The output cylinder produces the highest no-load output velocity of 62.2 mm/s and a maximum work pressure of 2.2 MPa [8].

However, the LDP has a low volume efficiency under certain pressure load. Switches of rectification valves produce step hydraulic load on the movers of the linear oscillation motor. For conventional feedback proportional-integral-derivative control, motor oscillation error increases with an increasing load pressure. This results in rectification disorder [9]. As a result, this discharges the chamber and absorbs the chamber, which gets short-circuited and the flow is consumed inside the pump.

In a conventional hybrid pump, due to the large driving stiffness of the piezoelectric motor, it is insensitive to the impact of the load. Therefore, their waveform distortion can be ignored. Research on piezoelectric control focuses on its hysteresis and other characteristics [10,11,12], and few studies pay attention to the modeling and compensation of the hydraulic load. Meanwhile, conventional application of the linear oscillating motor is driving a linear compressor. Their load is small and they have no phase control requirements. Therefore, in view of the working condition of the linear oscillating motor in the electro-hydrostatic actuator, a control method which can realize the compound control of amplitude and phase is needed.

Oscillation tracking accuracy is affected by nonlinear behavior, modeling uncertainties and unmeasured hydraulic load in the LDP system. The adaptive control is used extensively to deal with modeling uncertainties. An adaptive backstepping control scheme is used in the position control of the electro-hydrostatic actuator [13]. Active disturbance rejection adaptive control via full state feedback [14,15] and sliding mode adaptive control [16] are developed for hydraulic servo systems. This paper uses adaptive control to handle modeling uncertainties. In general, tracking trajectories of the linear motor in the LDP are periodic harmonic instructions, which is the same for the hydraulic load. For this kind of tracking command, repetitive control was developed [17], and its stability analysis was conducted theoretically [18]. However, a conventional repetitive learning algorithm is designed to adapt to all the values of periodic uncertainties in a cycle, which results in two problems in practice. First, the noise of the sensors can accumulate in the system which is mixed in command and reduces the control accuracy. Second, the recording of the periodic signal needs a lot of memory. In [19,20], Yao and Tomizuka developed the adaptive robust control (ARC). Based on ARC, we developed adaptive robust repetitive control schemes that solve the problematic heavy memory requirements and noise sensitivity [21,22].

This paper aims to apply the robust adaptive repetitive control to handle model uncertainties and step load in the linear oscillating motor. We did not measure the hydraulic load through sensors, which is used in some hydraulic servo controllers. The periodic hydraulic step is approximated by Fourier series, whose coefficients along with other model uncertainties are adapted by a discontinuous projection. The uncompensated nonlinearities are attenuated by certain robust control laws. Without extra sensors, the step load is compensated and a prescribed tracking accuracy can be achieved for sinusoidal trajectories up to 28 Hz. The rectification order recovers, and the volumetric efficiency is improved substantially.

The rest of this paper is organized as follows. Section 2 gives the structure of the LDP and its dynamic, and the hydraulic models are presented. Based on the model, the hydraulic step load is broken up into Fourier series and the adaptive repetitive controller is described in Section 3. Section 4 provides the test rig design and comparative experimental results on a linear oscillating motor control. Finally, a conclusion is presented in Section 5.

## 2. Problem Formulation and Dynamic Models

**Figure 1:**
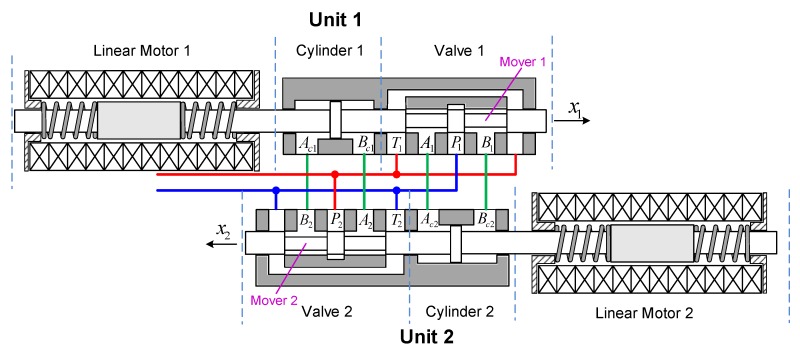
Schematic structure of the linear drive collaborative rectification structure pump (LDP).

As shown in Figure 1, the LDP consists of two identical units, and each of them has three parts. The first part is the linear oscillating motor. The second part is the cylinder, which has two chambers. The third part is a spool valve working as a rectification valve. Movers of three parts are linked together and driven by the linear oscillating motor for the linear reciprocating harmonic motion. The pressure difference of the two chambers in a unit reverses as the spool valve in the other unit switches the hydraulic circuit. Periodic changes result in a periodic step load on the linear oscillating motor. The goal is to estimate and compensate the step load and handle the nonlinearities and uncertainties. The dynamic of the mover can be described by

(1) $$\begin{array}{c} \left\{\begin{array}{cc} m{\ddot{y}}_{1} & =F_{m1}-B_{1}{\dot{y}}_{1}-K_{s}y_{1}-A_{f}S_{f}\left({\dot{y}}_{1}\right)-P_{L1}A\\ m{\ddot{y}}_{2} & =F_{m2}-B_{2}{\dot{y}}_{2}-K_{s}y_{2}-A_{f}S_{f}\left({\dot{y}}_{2}\right)-P_{L2}A \end{array}\right. \end{array}$$

where *m* is the mass of the mover, $y_{1},\;y_{2}$ are displacements of movers, $F_{m1},\;F_{m2}$ are driving forces of two motors, $B_{1},\;B_{2}$ are viscous damping coefficients of two units, $A_{f}S_{f}$ represents the approximated nonlinear Coulomb friction, of which $A_{f}$ is the Coulomb friction amplitude and $S_{f}$ is a known continuous shape function, $K_{s}$ is the elasticity coefficient of two parallel springs, *A* is the ram area of pistons and $P_{L1},\;P_{L2}$ are pressure differences of two chambers, i.e.,

(2) $$\begin{array}{c} \left\{\begin{array}{c} P_{L1}=P_{1a}-P_{1b}\\ P_{L2}=P_{2a}-P_{2b} \end{array}\right. \end{array}$$

where $P_{1a},\;P_{1b},\;P_{2a},\;P_{2b}$ are pressures of the forward and return chambers in unit 1 and unit 2. Thrusts of the motors $F_{m1},\;F_{m2}$ are also proportional to winding currents $i_{1},i_{2}$, i.e., [7]

(3) $$\begin{array}{c} \left\{\begin{array}{c} F_{m1}=K_{e}i_{1}\\ F_{m2}=K_{e}i_{2} \end{array}\right. \end{array}$$

where $k_{e}$ is the force to current coefficient. Current input of linear motor is directly proportional to the control voltage, i.e.,

(4) $$\begin{array}{c} \left\{\begin{array}{c} i_{1}=K_{d}u_{1}\\ i_{2}=K_{d}u_{2} \end{array}\right. \end{array}$$

where $i_{1}$ and $i_{2}$ are currents of the windings, $K_{d}$ is the amplification coefficient, and $u_{1}$ and $u_{2}$ are control outputs. Considering two units have the same dynamics, only unit 1 is analyzed in the following analysis. The pressure dynamics of cylinder chambers can be written as [23]

(5) $$\begin{array}{c} \left\{\begin{array}{cc} {\dot{P}}_{1a} & =\frac{\beta_{e}}{V_{0a}-Ay_{1}}\left(A{\dot{y}}_{1}-C_{t}P_{L1}+Q_{1a}\right)\\ {\dot{P}}_{1b} & =\frac{\beta_{e}}{V_{0b}+Ay_{1}}\left(-A{\dot{y}}_{1}+C_{t}P_{L1}-Q_{1b}\right) \end{array}\right. \end{array}$$

where $\beta_{e}$ is the effective bulk modulus, $V_{0a}$ and $V_{0b}$ are initial volumes in two chambers, $C_{t}$ is the coefficient of the internal leakage between two chambers, $Q_{1a}$ and $Q_{1b}$ are the supplied flow rate of the forward chamber and the return flow rate of the return chamber. $Q_{1a}$ and $Q_{1b}$ can be written as

(6) $$\begin{array}{c} \left\{\begin{array}{cc} Q_{1a} & =k_{q}\left|y_{2}\right|\left[\mathrm{sign}\left(P_{s}-P_{1a}\right)s\left(y_{2}\right)\sqrt{|P_{s}-P_{1a}|}+\mathrm{sign}\left(P_{r}-P_{1a}\right)s\left(-y_{2}\right)\sqrt{|P_{r}-P_{1a}|}\right]\\ Q_{1b} & =k_{q}\left|y_{2}\right|\left[\mathrm{sign}\left(P_{1b}-P_{r}\right)s\left(y_{2}\right)\sqrt{|P_{1b}-P_{r}|}+\mathrm{sign}\left(P_{1b}-P_{s}\right)s\left(-y_{2}\right)\sqrt{|P_{1b}-P_{s}|}\right] \end{array}\right. \end{array}$$

where $k_{q}$ and s() are defined as

(7) $$\begin{array}{c} k_{q}=C_{d}w\sqrt{2/\rho } \end{array}$$

(8) $$\begin{array}{c} s\left(x\right)=\left\{\begin{array}{cc} 1,\;\;\;\mathrm{if} & x\geq 0\\ 0,\;\;\;\mathrm{if} & x<0 \end{array}\right. \end{array}$$

where $P_{s}$ and $P_{r}$ are the output pressure of the pump and the suction pressure, and both are constant. $C_{d}$ is the discharge coefficient, *w* is the equivalent spool valve area gradient and ρ is the fluid density.

The pressure dynamic of chambers is complicated. Obviously, the unit of the LDP system is not a semi-strict feedback system. Moreover, the displacement of one unit is an input of the other unit which forms a coupling relation. Lacking enough state observers, the precise control of the linear oscillating motor is hard to achieve without the repetitive control method. With reasonable analysis, in the steady state of LDP, $y_{1}$ and $y_{2}$ are periodic. Considering Equations (5) and (6), the variables all are periodic. Flow rates into and out of chambers and pressures in chambers are periodic, and then hydraulic loads $P_{L1}A$ and $P_{L2}A$ are periodic. They change in periods of *T*, i.e.,

(9) $$\begin{array}{c} \left\{\begin{array}{c} P_{L1}\left(t+T\right)=P_{L1}\left(t\right)\\ P_{L2}\left(t+T\right)=P_{L2}\left(t\right) \end{array}\right. \end{array}$$

Define the system state vector ${\left[x_{1},x_{2}\right]}^{T}={\left[y_{1},{\dot{y}}_{1}\right]}^{T}$, and then the simplified dynamics of the system is given by

(10) $$\begin{array}{c} \begin{array}{cc} {\dot{x}}_{1} & =x_{2}\\ m{\dot{x}}_{2} & =K_{m}u-B_{1}x_{2}-K_{s}x_{1}-A_{f}S_{f}\left(x_{2}\right)-P_{L1}A \end{array} \end{array}$$

where

(11) $$K_{m}=K_{e}K_{d}$$

The desired displacement command of motor 1 $x_{1d}=y_{1d}$ is a sinusoidal instruction. It is third-order continuous and differentiable.

## 3. Nonlinear Adaptive Repetitive Controller Design

### 3.1. Design Model and Issues to be Addressed

In Equation (10), the term $P_{L1}A$ is highly periodic with a known period *T*. It can be represented by a finite Fourier series

(12) $$P_{L1}=\frac{A_{0\alpha }}{2}+\sum_{n=1}^{m}\left(A_{n\alpha }\cos n\omega t+B_{n\alpha }\sin n\omega t\right)+\Delta_{\alpha }=\varphi_{d}^{T}\theta_{d\alpha }+\Delta_{\alpha }$$

where $\omega =\frac{2\pi }{T}$, $\theta_{d\alpha }=\left[\frac{A_{0\alpha }}{2},A_{1\alpha },B_{1\alpha },\cdots ,A_{m\alpha },B_{m\alpha }\right]$ represent the unknown Fourier coefficient, $\varphi_{d}^{T}=\left[1,\cos\omega t,\sin\omega t,\cdots ,\cos m\omega t,\sin m\omega t\right]$ are the basis functions and δ is the unknown variation between the series and the true nonlinearity. Considering that general physical mechanical systems are low-pass filters with finite bandwidth, the first few terms will be enough for a good approximation in practice. Moreover, the system is subjected to structured uncertainties due to large variations in the hydraulic parameters, typically, the variations of $B_{1},\;K_{s}$ and $A_{f}$ due to the change of temperature and component wear. In order to simplify the state–space equation, we define the unknown parameter set $\theta_{\alpha }^{T}=\left[\theta_{1\alpha },\theta_{2\alpha },\theta_{3\alpha },\theta_{d\alpha }^{T}\right]$ with $\theta_{1\alpha }=B_{1}$, $\theta_{2\alpha }=K_{s}$ and $\theta_{3}=A_{f}$. The state–space Equation (10) is now linearly parameterized in terms of $\theta_{\alpha }$ as

(13) $$\begin{array}{c} \begin{array}{cc} {\dot{x}}_{1} & =x_{2}\\ m{\dot{x}}_{2} & =K_{m}u-\theta_{1\alpha }x_{2}-\theta_{2\alpha }x_{1}-\theta_{3\alpha }S_{f}\left(x_{2}\right)-\varphi_{d}^{T}\theta_{d\alpha }+\Delta_{\alpha } \end{array} \end{array}$$

The values of parameters in set $\theta_{\alpha }$ are not known. However, the range of it is known by analysis and experience. Thus, we can make the following reasonable and practical assumption of the parameters:

**Assumption** **1.** *The extent of parametric uncertainties and uncertain nonlinearities is known, i.e.,*

(14) $$\begin{array}{c} \begin{array}{cc} \; & \theta_{\alpha }\in \Omega_{\theta }≜\left\{\theta_{\alpha }:\theta_{\alpha \min}<\theta_{\alpha }<\theta_{\alpha \max}\right\},\\ \; & \Delta_{\alpha }\in \Omega_{\Delta }≜\left\{\Delta_{\alpha }\left|\right|\Delta_{\alpha }\left(x,t\right)\left|\right|\leq \delta_{\alpha }\left(x,t\right)\right\}, \end{array} \end{array}$$

*where $\theta_{\alpha \min}$, $\theta_{\alpha \max}$, and $\delta_{\alpha }\left(x,t\right)$ are known.*

### 3.2. Projection Mapping and Parameter Adaptation

We introduce ${\hat{\theta }}_{\alpha }$ to represent the estimate of $\theta_{\alpha }$, and ${\tilde{\theta }}_{\alpha }$ to represent the estimation error (i.e., ${\tilde{\theta }}_{\alpha }={\hat{\theta }}_{\alpha }-\theta_{\alpha }$). Under Assumption 1, the idea of a discontinuous projection based ARC design can be applied to solve the tracking control problem for Equation (10). Specifically, if the initial estimate satisfies the physical constraints Equation (14), the following “learning” algorithm is applied to update the estimate

(15) $${\dot{\hat{\theta }}}_{\alpha }={\mathrm{Proj}}_{{\hat{\theta }}_{\alpha }}\left(\Gamma \tau \right)$$

where Γ is any symmetric positive definite adaptation rate matrix. Specifically, Γ is assumed to be a diagonal matrix in the sequel. τ is an adaptation function to be specified later, and the projection mapping ${\mathrm{Proj}}_{{\hat{\theta }}_{\alpha }}\left(•\right)$ is defined by [24]

(16) $$\begin{array}{c} {\mathrm{Proj}}_{{\hat{\theta }}_{\alpha }}\left({•}_{i}\right)=\left\{\begin{array}{cc} 0,\; & if\;{\hat{\theta }}_{\alpha i}=\theta_{\alpha i\max}\;and\;{•}_{i}>0\\ 0, & if\;{\hat{\theta }}_{\alpha i}=\theta_{\alpha i\min}\;and\;{•}_{i}<0\\ {•}_{i}, & otherwise \end{array}\right. \end{array}$$

where ${•}_{i}$ represents the *i*th component of the vector •. As proven in [24,25], the adaptive algorithm built by Equations (15) and (16) has the following properties:

(17) $$P1.\;{\hat{\theta }}_{\alpha }\in \Omega_{\theta }≜\left\{{\hat{\theta }}_{\alpha }:\theta_{\alpha \min}<{\hat{\theta }}_{\alpha }<\theta_{\alpha \max}\right\}$$

(18) $$P2.\;{\hat{\theta }}_{\alpha }^{T}\left[\Gamma^{-1}{\mathrm{Proj}}_{{\hat{\theta }}_{\alpha }}\left(\Gamma \tau \right)-\tau \right]\leq 0\;\forall \tau$$

### 3.3. Controller Design

We first define the tracking error as $z_{1}=x_{1}-x_{1d}$ and the filtered errors as

(19) $$\begin{array}{c} z_{2}={\dot{z}}_{1}+k_{1}z_{1}=x_{2}-x_{2eq},\;x_{2eq}≜{\dot{x}}_{1d}-k_{1}z_{1} \end{array}$$

where $k_{1}$ is a positive feedback gain. Differentiating $z_{2}$, we have

(20) $$\begin{array}{cc} m{\dot{z}}_{2} & =K_{m}u-\theta_{1\alpha }x_{2}-\theta_{2\alpha }x_{1}-\theta_{3\alpha }S_{f}\left(x_{2}\right)-\varphi_{d}^{T}\theta_{d\alpha }+\Delta_{\alpha }-m{\dot{x}}_{2eq}\\ \; & =K_{m}u+l\varphi^{T}\theta_{\alpha }+\Delta_{\alpha }-m{\dot{x}}_{2eq} \end{array}$$

where $\varphi^{T}=\left[-x_{2},-x_{1},\;-S_{f}\left(x_{2}\right),\;-\varphi_{d}^{T}\right]$. The ARC control law is given by

(21) $$\begin{array}{cc} \; & v\left(x_{1},x_{2},\hat{\theta },t\right)=v_{a}+v_{s},\;v_{a}=m{\dot{x}}_{2eq}+{\hat{\theta }}_{1\alpha }x_{2}+{\hat{\theta }}_{2\alpha }S_{f}\left(x_{2}\right)+{\hat{\theta }}_{3\alpha }x_{1}+\varphi_{d}^{T}{\hat{\theta }}_{d\alpha }\\ \; & v_{s}=v_{s1}+v_{s2},\;v_{s1}=-k_{s1}z_{2} \end{array}$$

There are two parts, where $v_{a}$ is the adjustable model compensation for achieving perfect tracking, and $v_{s}$ is the robust control law consisting of two parts: $v_{s1}$ is a simple proportional feedback used to stabilize the nominal system and $v_{s2}$ is a robust feedback used to attenuate the effect of model uncertainties. $k_{s1}>0$ is a control parameter which need to be adjusted along with $k_{1}$, then we make the following matrix Λ positive definite

(22) $$\begin{array}{c} \Lambda =\left[\begin{array}{cc} k_{1} & -\frac{1}{2}\\ -\frac{1}{2} & k_{s1} \end{array}\right] \end{array}$$

Substituting Equation (21) into Equation (20), yields

(23) $$\begin{array}{c} m{\dot{z}}_{2}=-k_{s1}z_{2}+v_{s2}-\varphi^{T}{\tilde{\theta }}_{\alpha }-\Delta_{\alpha } \end{array}$$

Two constraints are given for $v_{s2}$

(24) $$C1.\;z_{2}\left[v_{s2}-\varphi^{T}\tilde{\theta }-\Delta_{\alpha }\right]\leq \varepsilon$$

(25) $$C2.\;v_{s2}z_{2}\leq 0$$

where ε is a positive design parameter representing the attenuation level of the model uncertainties. Constraint C1 in Equation (24) is used to represent the fact that $v_{s2}$ is synthesized to dominate the model uncertainties coming from both parametric uncertainties and unmodeled nonlinearities to achieve the guaranteed attenuation level ε. The passive-like C2 in Equation (25) is imposed to ensure that introducing $v_{s2}$ does not interfere with the nominal parameter adaptation process. A simple practice of $v_{s2}$ is given

(26) $$\begin{array}{c} v_{s2}=-\frac{1}{4\varepsilon }h^{2}z_{2} \end{array}$$

where $h\geq ∥\theta_{\alpha max}-\theta_{\alpha min}∥∥\varphi ∥+\delta \left(x,t\right)$. The $v_{s2}$ in Equation (26) is easy to compute in practice and is applied in the experiment shown in Section 4.

### 3.4. Main Results

The ARC control law has the following advantages:

**Theorem** **1.** *With the projection type adaptation law Equation (15) and adaptation function*

(27) $$\begin{array}{c} \tau =\varphi \left(x\right)z_{2} \end{array}$$

*The proposed ARC law Equation (21) guarantees that*

*A. In general, all signals are bounded, and the tracking error is bounded by*

(28) $$\begin{array}{c} |z_{1}|\leq \sqrt{2\exp\left(-\mu t\right)V\left(0\right)+\frac{2\varepsilon }{\mu }\left[1-\exp(-\mu t)\right]} \end{array}$$

*where V(0) is the initial value of the positive definite function V, which is defined by*

(29) $$\begin{array}{c} V=\frac{1}{2}z_{1}^{2}+\frac{1}{2}mz_{2}^{2} \end{array}$$

*where*

(30) $$\begin{array}{c} \mu =2\lambda_{\min}\left(\Lambda \right)\min\left\{1/m,1\right\} \end{array}$$

*is the exponentially converging rate, and the size of the final tracking error $\left(|z_{1}\left(\infty \right)|\leq \frac{2\varepsilon }{\mu }\right)$ can be freely adjusted by the controller parameters ε, $k_{1}$ and $k_{s1}$ in a known form.*

*B. If after a finite time $t_{0}$, there exist parametric uncertainties only (i.e., $\;\Delta \left(x,t\right)=0,\;\forall t⩾t_{0})$, then in addition to the results in A., an asymptotic output tracking is achieved, i.e., $z_{1}\to 0$ as t→∞.*

**Proof.**  Differentiating the positive definite function *V* while noting constraint C1 in Equation (24), we have

(31) $$\begin{array}{cc} \dot{V} & =z_{1}z_{2}-k_{1}z_{1}^{2}+k_{s1}z_{2}^{2}+z_{2}\left(v_{s2}-\varphi^{T}\tilde{\theta }-\Delta_{\alpha }\right)\\ \; & \leq -z^{T}\Lambda z+\varepsilon \\ \; & \leq -\lambda_{\min}\left(\Lambda \right)\left(z_{1}^{2}+z_{2}^{2}\right)+\varepsilon \end{array}$$

where $\lambda_{\min}\left(\Lambda \right)$ is the minimal eigenvalue of matrix Λ. Combining the definition of μ in Equation (30), yields

(32) $$\begin{array}{c} \dot{V}\leq \varepsilon -\mu V \end{array}$$

Therefore, using the comparison lemma [26], we can obtain

(33) $$\begin{array}{c} V\leq \exp\left(-\mu t\right)V\left(0\right)+\frac{\varepsilon }{\mu }\left[1-\exp(-\mu t)\right] \end{array}$$

Substituting Equation (29) back leads to part A.

Now consider the situation in B. of Theorem 1 (i.e., $\Delta_{\alpha }=0$). Define another positive definite function $V_{a}=V+\frac{1}{2}{\tilde{\theta }}^{T}\Gamma^{-1}\tilde{\theta }$, whose derivative is

(34) $$\begin{array}{c} {\dot{V}}_{a}=z_{1}z_{2}-k_{1}z_{1}^{2}+k_{s1}z_{2}^{2}+z_{2}\left(v_{s2}-\varphi^{T}\tilde{\theta }\right)+{\tilde{\theta }}^{T}\Gamma^{-1}\dot{\hat{\theta }} \end{array}$$

Noting constraint Equation (25) and the adaptation function Equation (27), we have

(35) $$\begin{array}{cc} {\dot{V}}_{a} & =-z^{T}\Lambda z+z_{2}v_{s2}+{\tilde{\theta }}^{T}\left(\Gamma^{-1}\dot{\hat{\theta }}+\varphi z_{2}\right)\\ \; & \leq -z^{T}\Lambda z+{\tilde{\theta }}^{T}\left(\Gamma^{-1}\dot{\hat{\theta }}+\tau \right) \end{array}$$

From P2, in Equation (18), we obtain

(36) $$\begin{array}{c} {\dot{V}}_{a}=-\lambda_{\min}\left(\Lambda \right)\left(z_{1}^{2}+z_{2}^{2}\right)≜-W \end{array}$$

This shows that $V_{a}\leq V_{a}\left(0\right)$. Therefore, $W\in L_{2}$ and $V_{s}\in L_{\infty }$. Since all signals are bounded, $\dot{W}$ is bounded in Equation (23). Thus, *W* is uniformly continuous. By Barbalat’s lemma, W→0 as t→∞, which leads to the asymptotic tracking in B. □

**Remark** **1.** *Results in A. of Theorem 1 indicates that the proposed controller has an exponentially convergent transient performance with the exponentially converging rate μ and the final tracking error is able to be adjusted via certain controller parameters freely in a known form. Part B. of Theorem 1 implies that the parametric uncertainties may be reduced through parameter adaptation and an asymptotic converging performance is obtained.*

## 4. Comparative Experimental Results

### 4.1. Experiment Setup

**Figure 2:**
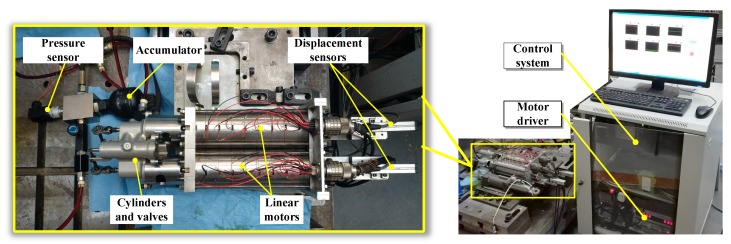
Experimental setup.

The LDP prototype and test rig are shown in Figure 2. Two linear oscillating motors were parallel fabricated. The rest of the components included two driving amplifier (AMC 50A20I), a two-cylinders–two-valves assembly, two displacement sensors, an accumulator, a pressure sensor, an oil tank and the measurement and control system. Key experimental parameters of the experiment components are listed in Table 1. The measurement and control system was made up of a real-time PXI controller (PXI 1036, National Instrument Corporation), an analog input module (PXI 6733, NI), an analog output module (PXI 6281, NI) and a host computer. The monitor and real-time control software are programmed with NI Labview. The sampling and control period was 1.2 ms.

### 4.2. Comparative Experimental Results

The sinusoidal trajectories have a certain frequency which is the resonant frequency of the linear motor, and its amplitude is limited by the compression of the springs. Thus, the sinusoidal trajectory is r(t)=sin(56πt). The following two controllers are compared.

**Figure 3:**
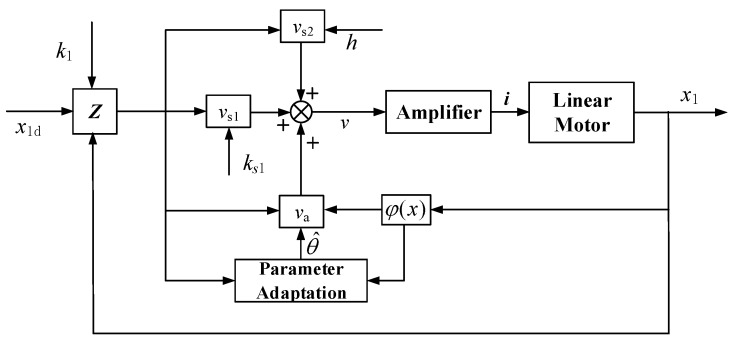
Block diagram of the adaptive robust control (ARC).

1. APC: The nonlinear adaptive repetitive controller in the above section. To simplify the controller and save memory, only a small number of unknown parameters are adapted, i.e., *m* in Equation (12) is set to 3. So $\varphi_{d}^{T}\theta_{d\alpha }$ and its counterpart of motor 2 $\varphi_{d}^{T}\theta_{d\beta }$ all have seven terms. The initial values for the parameters are set to ${\hat{\theta }}_{\alpha }\left(0\right)={\left[70,36000,5,0,-150,10,5,5,35,15\right]}^{T}$ and ${\hat{\theta }}_{\beta }\left(0\right)={\left[70,36000,5,0,115,115,35,35,15,15\right]}^{T}$. Viscous damping coefficients $B_{1}$ and $B_{2}$ as well as the Coulomb friction amplitude $A_{f}$ were identified by Wang in the design process of the linear oscillating motor [27]. Even if the wear of linear bears and different working conditions change the true values of parameters slightly, it is reasonable to set identified values as the initial values. $K_{s}$ is the elasticity coefficient of two parallel springs. Its initial value was set to its nominal value. The initial value of the load pressure of the pump was set to 2.8 MPa by adjusting the throttle valve. The hydraulic load of linear motor is periodic. Its initial amplitude can be approximated as the product of load pressure and ram area of piston and its phase is synchronous with displacement of the other linear motor. Initial Fourier coefficients were got by applying Fourier transformation to the periodic initial hydraulic load. The bounds of the parameter variations in the two motor systems are the same and are estimated as $\theta_{min}={\left[60,30000,-20,-50,-260,-260,-260,-260,-130,-130\right]}^{T}$ and $\theta_{max}={\left[100,40000,20,50,260,260,260,260,130,130\right]}^{T}$. The magnitude of Δ is assumed to be less than 500, i.e., $\delta {\left(x,t\right)}_{\alpha }\leq 500$ and $\delta {\left(x,t\right)}_{\beta }\leq 500$. The control gains in two motors are the same: $k_{1}=20$, $k_{s1}=600$. The shape function of Coulomb friction $S_{f}\left(x_{2}\right)=\arctan\left(1000x_{2}\right)$ [21]. Adaptation rate matrix Γ in the two systems are identically set: Γ=diag[50,5,100,100,100,100,100,100,10,10]. The control diagram is shown in Figure 3.
2. Proportional-integral-derivative (PID): A conventional proportional-integral-derivative controller was built. We optimized the PID parameters in experiments. Derivative section is sensitive to interference, whose coefficient was set to zero. Considering the working condition of the LDP, we paid attention to the steady state response of the linear motor, when we optimized PI coefficients. The hydraulic pressure of 2.8 MPa was applied on the LDP. Because of coupling effect of two motors, the amplitude attenuation and phase lag of the single linear motor cannot reflect the control performance. The output flow rate of LDP was measured and recorded, which was used as the optimization objective. We got the optimal PI coefficients when the flow rate was maximum. The controller parameters are $k_{\alpha p}=65,\;k_{\alpha i}=89,\;k_{\alpha d}=0$, $k_{\beta p}=65,\;k_{\beta i}=89,\;k_{\beta d}=0$, which represent the P-gain, I-gain and D-gain of two motors, respectively.

To quantify the performance of our controller, the following performance indices are used [28]:

1. Maximal absolute value of the tracking errors is defined as
  (37) $$\begin{array}{c} M_{e}=\max_{i=1,\ldots ,N}|z_{1}\left(i\right)| \end{array}$$
  where *N* is number of the recorded digital signals, and is used as an index of measure of tracking accuracy.
2. Average tracking error is defined as
  (38) $$\begin{array}{c} \kappa =\frac{1}{N}\sum_{I=1}^{n}|z_{1}\left(i\right)| \end{array}$$
  which is used as a measure of average tracking performance.
3. Standard deviation performance index is defined as
  (39) $$\begin{array}{c} \sigma =\sqrt{\frac{1}{N}\sum_{I=1}^{n}{\left(|z_{1}\left(i\right)|-\kappa \right)}^{2}} \end{array}$$
  to measure the deviation level of tracking errors.

**Figure 4:**
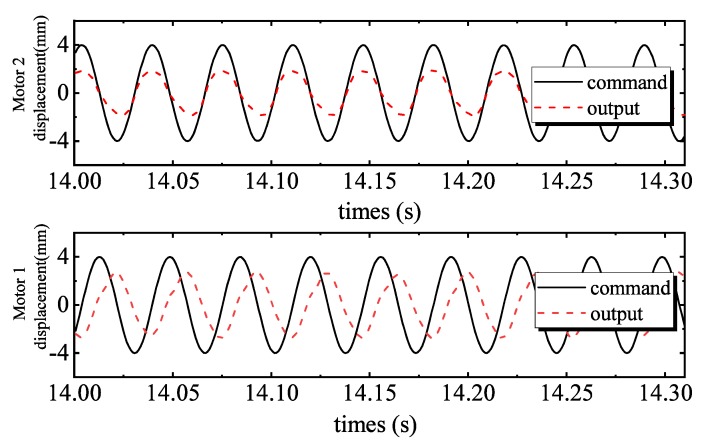
Proportional-integral-derivative (PID) tracking performance of two motors.

**Figure 5:**
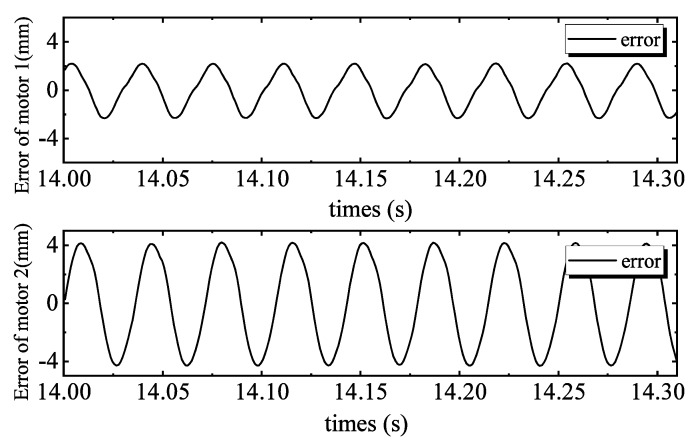
PID tracking errors of two motors.

**Figure 6:**
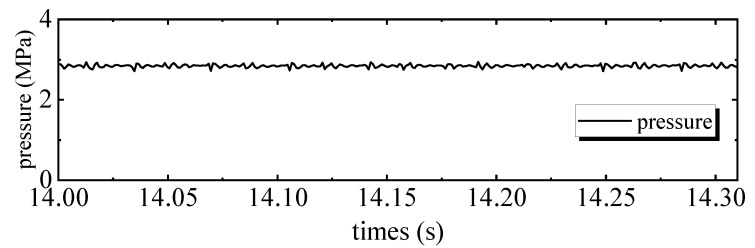
Load pressure of the linear drive collaborative rectification structure pump of PID.

**Figure 7:**
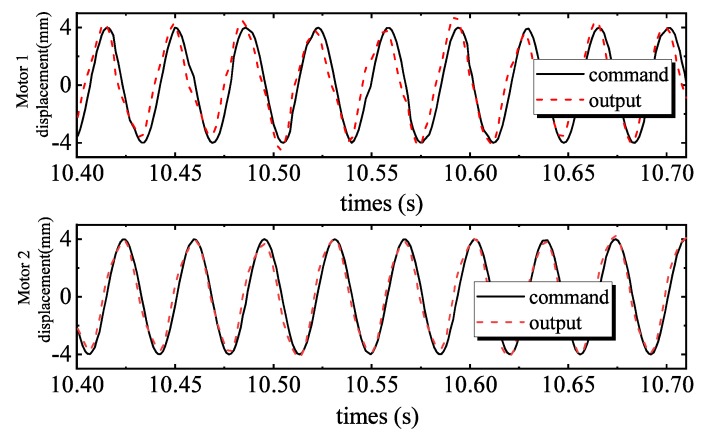
ARC tracking performance of two motors.

**Figure 8:**
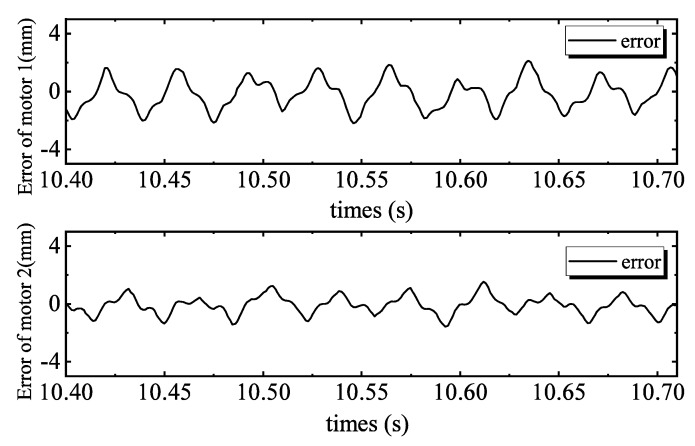
ARC tracking errors of two motors.

**Figure 9:**
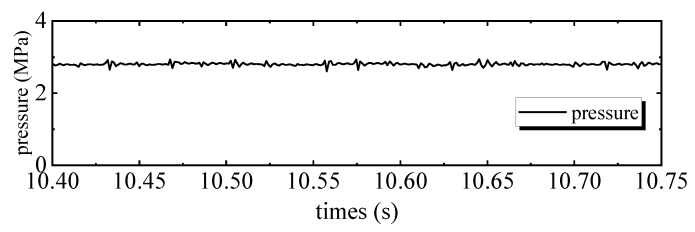
Load pressure of the linear drive collaborative rectification structure pump of ARC.

Figure 4 shows the tracking performance of the PID controller. Tracking errors are illustrated in Figure 5. Quantified performances are shown in Table 2. The tracking performance is bad and the maximum error of motor 2 is 4.3039 mm, which is bigger than the total travel. In the 28 Hz operating frequency, unmodeled dynamics such as the structural stiffness and joint clearances have more affect on outputs, compared to the low frequency tracking work. Moreover, the hydraulic step load of 2.8 MPa (shown in Figure 6) degrades amplitude and phase responses. Figure 7 and Figure 8 show the tracking performance and tracking error of APC, respectively. The hydraulic load is 2.8 MPa (shown in Figure 9) as well. According to these experimental results shown in Table 2, it can be seen that the proposed APC controller outperforms the PID controller in terms of all performance indices. This illustrates the effectiveness of the proposed scheme with appropriate compensation of the periodic hydraulic step load.

The experimental results show the ARC control did not achieve ideal tracking accuracy, and the theoretical control results in Section 3 are not verified fully. There are two reasons. First, in the 28 Hz oscillating frequency, the dynamic of the motor amplifier becomes effective. There is lag between the control command and armature current. Second, in the high operating frequency, unmodeled dynamics such as the structural stiffness and joint clearances take effect. These problems need more studies in the future.

## 5. Conclusions

In this paper, a nonlinear adaptive repetitive controller is proposed via a Fourier series approximation for a pair of linear oscillating motors. The control scheme applies Fourier series to approximate the periodic hydraulic step load of the linear drive collaborative rectification structure pump. The series’ coefficients, as well as other uncertain parameters, are estimated and compensated by the adaptive control. A robust control term is added to deal with uncertain nonlinearities. The controller can achieve a prescribed final tracking accuracy under periodic hydraulic step load via Lyapunov analysis. Comparative experimental results from the tracking control of sinusoidal trajectories up to 28 Hz show that the adaptive repetitive controller has a better performance. Meanwhile, the room for improvement of the controller is revealed. The amplifier’s dynamics and other structure dynamics at a high frequency need more accurate modeling and compensating in the future.

## Figures and Tables

**Table 1:** Key experimental parameters of test system.

Items	Symbol	Value
Stroke of cylinder	*S*	4 mm
Resonant frequency of linear motor	*f*	28 Hz
ram area of piston	$A_{p}$	48 ${\mathrm{mm}}^{2}$
Force coefficient of linear motor	$K_{e}$	27.5 N/A
Motor driver amplification coefficient	$K_{d}$	8 A/V
Mass of the mover	*m*	1.15 kg
Additive elasticity coefficient of two parallel springs	$K_{s}$	36,000 N/m

**Table 2:** Performance indices.

Indices	$M_{e}$	κ	σ
Motor 1 with PID	2.3219	1.3354	0.7263
Motor 2 with PID	4.3039	2.7644	1.2643
Motor 1 with APC	2.1675	0.8506	0.6028
Motor 2 with APC	1.5320	0.4887	0.3950

## Abbreviations

The following abbreviations are used in this manuscript:

LDP	Linear drive collaborative rectification structure pump
ARC	Adaptive robust control
PID	Proportional-integral-derivative
RISE	Robust integral of the sign of the error
